# Cost–Benefit Analysis of Real-Time Influenza Testing for Patients in German Emergency Rooms

**DOI:** 10.3390/ijerph16132368

**Published:** 2019-07-03

**Authors:** Roland Diel, Albert Nienhaus

**Affiliations:** 1 Institute for Epidemiology, University Medical Hospital Schleswig-Holstein, Kiel, Airway Research Center North (ARCN), 24015 Kiel, Germany; 2 Lung Clinic Grosshansdorf, Germany. Airway Disease Center North (ARCN), German Center for Lung Research (DZL), 22949 Großhansdorf, Germany; 3 Institution for Statutory Accident Insurance and Prevention in the Health and Welfare Services (BGW), 22089 Hamburg, Germany; 4 Institute for Health Service Research in Dermatology and Nursing, University Medical Center Hamburg-Eppendorf, 20246 Hamburg, Germany

**Keywords:** cost–benefit analysis, sensitivity analysis, PCR, real-time testing, influenza

## Abstract

Background: Seasonal influenza causes significant morbidity worldwide and has a substantial economic impact on the healthcare system. Objective: To assess the cost–benefit relation of implementing a real-time influenza test in emergency rooms (ER) of German hospitals. Methods: A deterministic decision-analytic model was developed simulating the incremental costs of using the Solana^®^ Influenza A+B test, compared to those of using conventional clinical judgement alone to confirm or exclude influenza in adult ILI (influenza-like illness) patients, in German ER, prior to hospitalization. Direct costs were evaluated from the hospital perspective, considering resource use directly related to influenza testing and treatment, as well as indirect costs incurred by nosocomial influenza transmission. Results: Through base-case analysis and assuming an influenza prevalence of 42.6%, real-time testing with Solana^®^ reduced average costs of hospitalized ILI patients by €132.61, per tested patient. Moreover, the Solana^®^ saved €6.9 per tested patient in favor of the hospital. In probabilistic sensitivity analysis, under all reasonable assumptions, implementing the Solana^®^ saved on average €144.13 as compared to applying the clinical-judgement-only strategy, thus, it was found to be constantly less expensive. Conclusions: Using highly sensitive and specific real-time influenza tests in ILI patients at German ER might significantly reduce hospital expenditures

## 1. Introduction

Seasonal influenza remains a worldwide, annually recurring public health challenge. In Germany, in the 2017/2018 season there were 334,000 confirmed influenza patients (nearly 400/100,000) [1] and the prevalence among influenza-like illness (ILI) patients was 42.6% [2]. Eighteen percent (18%) of those patients—with no difference between the proportion of ILI patients and patients with laboratory confirmed influenza—had to be hospitalized, imposing a high economic burden to the statutory public health insurances (PHI) [3]. However, as all inpatients with influenza have to be kept in costly respiratory isolation until it can be assumed that they are no longer contagious, a correct classification of the ILI as being truly caused by the influenza virus, rather than other viruses, e.g., parainfluenza virus, respiratory syncytial virus (RSV), or adenoviruses, helps to a great extent before making the expensive decision to isolate. 

Furthermore, rapid diagnosis of influenza is essential not only for early treatment and the associated prevention of transmission of the virus, but it is also highly relevant to the management of scarce economic resources. Since 1 January 2004, hospital costs in Germany are based on the German diagnosis-related groups (G-DRG) system, which assigns each influenza to one of two categories (D62Z or E79D), depending on the presence of comorbidities (pneumonia, exacerbating COPD, or bronchiectasis, etc.). This imposes a fixed “base rate” of payment for 7 or 13 days of treatment. If the hospital treatment exceeds the so-called “mean length of stay”, i.e., 3.5 (category D62Z) and 6.7 (category E79D) days (as calculated mathematically by the DRG Institute for Hospital Reimbursement (InEK)), then the G-DRG rate paid as reimbursement by the statutory health insurances usually does not cover the costs incurred by the hospital. Accordingly, in treating influenza patients covered by the statutory health insurance, hospitals should try to keep the duration of hospital stays as short as possible [4]. 

Rapid influenza diagnostic tests (RIDTs)—immunoassays that detect viral antigens—have been used for diagnosis in influenza suspects in hospital emergency rooms (ER), for many years. Their heretofore sensitivity in the range of 50% to 70% [5] has limited their utility. In contrast, PCR influenza assays, which have near perfect sensitivity and specificity, are often performed in batches in clinical laboratories outside the hospital, necessitating specimen transport and—depending on the logistics of the respective lab—usually require a time-lag of at least one day before the report of the test result becomes available. Thus, in Germany, where currently 81.4% of all hospitals have eliminated their in-house laboratories [6], nasopharyngeal swabs or other respiratory specimen must usually be sent to external labs for PCR testing.

Recently, a new generation of easy-to-perform assays for rapid influenza testing based on isothermal amplification of nucleic acids with a sensitivity and specificity exceeding 95% (as compared to that of the best available reference standard, which is the centralized PCR assays) have been developed [7,8]. As their turnaround time (TAT) is less than 1 hour, these tests allow decentralized near-to-patient testing in ER.

Thus, our aim was to examine whether routine implementation of this new category of tests might lead to directly measurable economic advantages. For our model, we used the economic and clinical performance characteristics of the Solana^®^ Influenza A+B (manufactured by Quidel Inc., San Diego, CA, USA), as performed in or within close proximity to emergency rooms (ER) of a hospital. We compared economic outcomes of this measure to those to be expected when conventional clinical judgement alone is used to confirm or exclude influenza in patients with influenza-like illness (ILI), who would subsequently need to be hospitalized. 

### 1.1. Test System 

The Solana^®^ Influenza A+B Assay (Quidel Inc.) is a qualitative isothermal NAT-amplification assay for the detection and differentiation of influenza A and influenza B viral RNA. A total of 50 µl of nasopharyngeal specimen received in the viral transport media is added to a labeled Process Buffer Tube and heat lysed for 5 min at 95 °C (specimen preparation). Subsequently, 50 µL of the heat-lysed specimen is transferred into a reaction tube and placed into the Solana^®^ device for target amplification and detection (amplification and detection). As testing with the Solana^®^ is designed to be performed by minimally trained personnel, and as the time for obtaining the influenza test result is about 45 minutes, the Solana^®^ is an easy-to-use rapid test. However, according to the definition of Luppa and Junker [9], it cannot be considered a point-of-care test in the strict sense, as there is a waiting time of about 5 minutes during heat lysis in the device and as two, manual, intermediate pipetting steps have to be performed. Testing results can be printed, stored, and transferred to the laboratory information system.

According to the manufacturer (personal communication), the costs per test of Solana^®^ are on average €14. The required instrument is provided on loan for an unlimited period and maintained regularly at no charge by the manufacturer, without obligation to pay extra charges on a later occasion.

### 1.2. Ethical Considerations 

Ethical approval was not necessary as only fully anonymized secondary data were used.

## 2. Materials and Methods 

### 2.1. Model Approach

Our model was parametrized by data on sensitivity and specificity of the Solana^®^ compared to the conventional clinical approach. After pipetting 50 µL of the vortexed nasopharyngeal swab for Solana^®^ testing, the remaining tube containing the viral transport medium could be sent to an external laboratory. The prevalence of influenza virus among ILI patients might differ from season to season; the most recent prevalence data for the influenza season period 2017/2018 were taken from the German RespVir-Net [2].

For the Solana^®^, two scenarios were considered. In the first, the Solana^®^ algorithm was used, in which all ILI patients coming to the ER of a hospital during a seasonal influenza pandemic are tested with the Solana^®^, after using a nasopharyngeal swab. Depending on the severity of symptoms, a patient would be hospitalized or discharged from the ER. In case of hospitalization, the patient would be isolated from the moment of presumptive diagnosis, given a positive Solana^®^ test result, through the first seven days of stay (or, upon resolution of fever and respiratory symptoms for an additional 24 hours). This is to counteract the nosocomial spread of influenza, according to U.S. [10] and German guidelines [11]. Given the high sensitivity and specificity of Solana^®^ (each over 95%, see below for details), additional PCR testing was not required. Under the premise that the patient arrived at the ER within the first 48 hours following the onset of his symptoms, an antiviral neuraminidase inhibitor (NI), Oseltamivir, would immediately be administered to shorten symptom duration by reducing the viral load [12,13,14]. 

In the alternative (versus Solana^®^) scenario, i.e., the conventional clinical approach, the decision as to whether the present ILI is caused by influenza would be taken through symptom-based judgement, without rapid pre-testing. Thus, if hospitalization were required, the decision to isolate an influenza suspect and to offer NI would only be based on that clinical decision. Due to the low sensitivity and specificity of clinical judgement, (see below for details) a patient sample in the form of a nasopharyngeal swab would be taken from those ILI patients judged as requiring hospitalization. These samples would be sent to an external laboratory for PCR testing that ideally has both a sensitivity and a specificity of 100%, irrespective of the result of the clinical judgement. This would clarify whether or not the disease is due to influenza and, accordingly, whether the patient requires isolation. In daily clinical practice, this (costly) procedure is usually not done for the mild cases in which a low viral load can be assumed and which are not considered to require hospitalization. 

If the patient were not to be hospitalized but discharged and sent home from the ER, the PHI would be charged for the Solana^®^ influenza test with the corresponding Einheitlicher Bewertungsmaßstab (EBM) figure Gebührenordnungsposition (GOP) 32791 via the Kassenärztliche Vereinigung (KV, Association of Statutory Health Insurance Physicians). Thus, no additional costs incur for those outpatients at the expense of the hospital, and the costs of Solana^®^ testing in those patients does not get included in our analysis. 

In contrast, the costs for rapid testing of those patients thar are ultimately hospitalized have to be paid by the hospital, and also the externally performed PCR would be directly billed to the hospital by the external laboratory, in accordance with the German Scale of Medical fees (GOÄ).

Additional costs from the hospital perspective are opportunity costs that might occur as long as an influenza suspect is uneccesarily kept in isolation (see details below). This occurs in the cases of false-positive clinical judgement or a false-positive rapid test. Under the premise that most influenza patients are accommodated in a twin-bedded room and that hospital wards in Germany during influenza season are working at full or nearly full capacity, the economic losses caused by blocking the second bed are incurred by the hospital itself.

If a patient is isolated due to erroneous clinical judgement (no influenza present), the isolation can be ended as soon as the report of the negative laboratory PCR result is available. It is assumed that administering Oseltamivir would be continued for 5 days [15,16] if influenza infection is confirmed by the external PCR or, if the PCR result is negative, it would be stopped immediately.

In most cases, patients can be discharged one day earlier than that forseen by the DRG, given the expected response to the Oseltamivir treatment [17]. As the hospital receives a fixed DRG flat rate, this results in an economic benefit to the hospital.

If the Solana^®^ is used as described here, a false-positive test result would not be corrected (like the clinical judgment would) after 1–2 days, because no external PCR test would be performed. Under the worst case assumptions, patients that falsely tested positive would end up being isolated for 7 days and ineffectively receive NI for 5 days. Early release (one day earlier) is, therefore, out of the question. 

Our model also includes the effects of transmission by undetected influenza patients to co-patients or healthcare workers (HCW) by incorporating a secondary attack rate. The measured effect is sick days for hospital workers, the costs of which, under the German system, is borne by the hospital. 

For patients who are sent home directly from the ER, the costs of routine diagnostics (chest X-ray, routine laboratory values, physical examination, etc.) as well as those of the rapid testing performed in the ER, are covered by the PHI and not the hospital, and are, therefore, outside the scope of our model.

### 2.2. Model Structure

**Figure 1:**
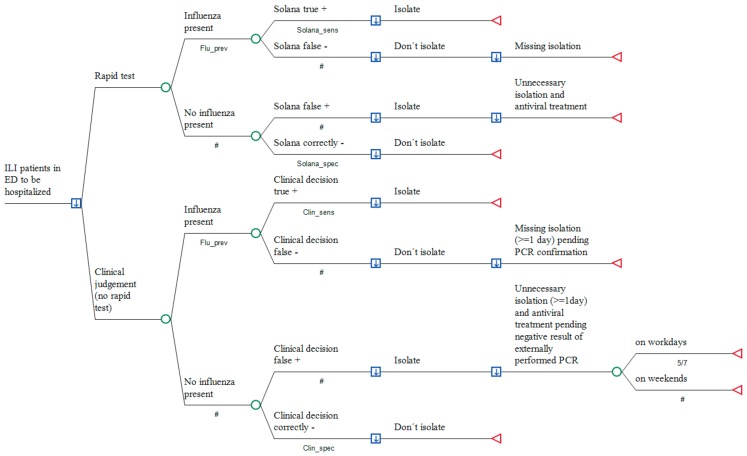
Real-time testing versus the conventional approach in influenza suspects prior to hospitalization. A decision node (square) indicates a choice facing the decision maker or the consequences of a decision. Branches from a change node (circles) represent the possible outcomes of an event; terminal nodes (triangles) denote the endpoints of a scenario and are assigned the cost of a prior series of actions and events. The arrows in the decision notes pointing downwards demonstrate that the optimal path of the model is that with the lowest total cost. #: Complementary probability (all probabilities of chance node’s branches to sum to 1.0); +: positive; −: negative.

A deterministic, patient-based, decision-analytic model was developed, simulating the incremental costs of using Solana^®^ in adult patients who attend the ER of a German hospital with acute moderate-to-severe respiratory infection and suspicion of influenza, compared to conventional clinical judgement. The perspective taken is that of the hospital itself (see Figure 1). 

Treatments compared were, as described above—(1) empiric clinical investigation without testing for viruses, or (2) real-time influenza testing used to guide antiviral treatment and the decision as to whether a patient—if hospitalization is required due to signs of severe lower respiratory infection—requires isolation. As real-time testing in those patients who will be discharged and sent home from ER is paid by the local KV and external PCR is not required in such mild cases, the decision tree is restricted to patients due for hospitalization.

Total costs of outcomes were simulated for each study arm, including (1) the medical cost of Solana^®^ testing and administering Oseltamivir; (2) medical costs of external PCR testing in all clinically judged patients without rapid testing prior to hospitalization; (3) opportunity costs due to blocking a twin-bed reimbursement per day of hospital stay within the fixed payment DRG period; (4) reimbursement per day of hospital stay within the fixed payment DRG period; (5) sick pay costs at the expense of the hospital if secondarily infected by hospitalized but unrecognized influenza patients; and (6) sick pay costs at the expense of the hospital if secondarily infected by hospitalized but unrecognized influenza patients. 

We used the TreeAge Software (TreeAge Inc. Williamstown MA, USA) for model building and analysis and examined our inputs over a wide range in sensitivity analyses to identify influential factors that would alter the base-case findings. First, univariate sensitivity analysis was performed using all variables to examine the extent to which our calculations were affected by the varying selected assumptions. Variation was done using either (a) the lower and upper bounds of a parameter´s standard deviation or (b) those of its 95% confidence interval. Where these are not applicable, our model simply causes parameter values to vary by ± 20% of the base-case value, according to international practice, unless stated otherwise. 

Furthermore, and in order to capture the interactions between multiple inputs, we provided a probabilistic sensitivity analyses (PSA) by assigning an appropriate statistical (probability) distribution for all parameters, randomly drawn in a second-order Monte–Carlo simulation (n = 10,000). All costs are reported in 2019 Euros (€). 

Input parameters are shown together with their probabilistic distributions in Table 1.

### 2.3. Model Input

#### 2.3.1. Epidemiological and Laboratory Parameters

##### Prevalence of Influence in Season 2017/2018

According to the data provided by the RespVir-net [2], there were approximately 55,500 findings of patients presenting flu-like symptoms during this period, of which 26,000 were virus-positive and 29,000 virus-negative. Of the 26,000 virus-positive findings, approximately (Flu A and Flu B taken together) 23,000 samples (= 88.5% of the virus-positive samples) were identified as influenza. In relation to all tests conducted on symptomatic patients (virus-positive + virus-negative), the rate of Flu-positive was 23,000/54,000 = 42.6% (95% CI.42.2–43.0%).

##### Sensitivity and Specificity of Clinical Judgement

As there are no gold criteria for a clinical case definition of ILI, widely varying values for sensitiv with ity and specificity of diagnosing influenza on the basis of clinical symptoms alone have been reported in multivariate analyses. The combination of fever plus cough rhinorrhea, sore throat, and headache in Yang´s South-Korean study [20] involving 1417 patients, during the influenza season 2011/2012, showed a sensitivity of 71.3% (CI 95%, 65.4%–76.7%) and a specificity of 60.1% (CI 95%, 57.2%–63%) versus laboratory confirmed influenza. Monto et al. [27], in their U.S. study of 3,744 patients with ILI symptoms, found sensitivity and specificity values of 64% and 67%, respectively (no confidence interval provided). In a prospective observational cohort study involving 78 adults admitted to hospital, clinical sensitivity and specificity of the clinical diagnosis was even lower, with only 60% and 53%, respectively [28]. We used the figures of Yang et al. as a baseline.

##### Sensitivity and Specificity of the Real-Time Influenza Test (Solana^®^)

According to a manufacturer´s publication [19], the sensitivity of the Solana^®^ is 97.2% (95% CI, 95.0%–98.4%) for Influenza A and 100% (95% CI, 95.4%–100%) for Influenza B. The specificity was 96.7 (95% CI, 95.4%–97.7%) for influenza A and 98.9% (95% CI, 98.2%–99.4%) for influenza (B), using an U.S. Food And Drug Administration (FDA) cleared influenza A+B molecular assay as the reference method. A value of combined sensitivity and specificity for influenza A and B, respectively, has not yet been provided. As the worst-case assumption, we took the respective lowest value (for detection of influenza A or B) as the combined value for sensitivity (97.2%) and specificity (96.7%) for our base-case calculations and the lower and upper values of the respective 95% CI, for the sensitivity analysis. 

##### Rate of Hospitalizations

According to the data of the survey “Bericht zur Epidemiologie der Influenza in Deutschland Saison 2017/2018” of the German Robert Koch Institute, the rate of hospitalization in the laboratory confirmed influenza cases was 18% (60,000/334,000, or 18.0%; 95% CI, 17.8%–18.1%) [1]. This was lower than the estimate of 23% for the season 2016/17 [29]. The lowest estimate was in season 2014/215 with 15.7% (11,000/70,000); in the season 2012/2013 the rate was 16.2% [30]. We took the value of 23% as the upper bound and the value of 15.7% as the lower bound for the sensitivity analysis. 

Of note, as explicitly mentioned in the Robert Koch Institute (RKI)-survey [1] and stated in the literature [31], the necessity of hospitalization depends on disease severity and can be considered the same for influenza and every other acute respiratory infection, during the influenza season. Accordingly, we used the value 18.1% as the base-case estimate for all influenza suspects.

##### Duration of Stay in Hospital by Antiviral Use

Aoki et al. [32] demonstrated the mathematical relationship between time for effective antiviral intervention and illness duration. Early administration of oral Oseltamivir corresponded to a benefit of ∼10 h (range 8–15) shorter duration of illness, for every 6 h earlier that the treatment was initiated. Thus, it seemed plausible that, as noted above, the duration of disease—and accordingly the absolute number of days spent in a hospital ward before being discharged—can be reduced by at least one day when drug administration is started quickly. 

##### Vaccination rate and its effectiveness among HCW 

Despite its known benefits, compliance with vaccination recommendations is low among health care personnel in Germany. According to a 2017 survey by the Robert Koch Institute, only 40.1% of all HCW have been vaccinated against influenza. The highest rate was that among physicians (61.4%), the lowest at 32.5%, among nurses [25]. However, vaccine effectiveness might vary, depending on how well the prevalent circulating viruses are matched to the influenza vaccine in the respective season. During the 2017/2018 season, as in the season 2015/2016, vaccine effectiveness in Germany, adjusted to age, gender, week of onset of the disease, and pre-existing illness, was only 15% (95% CI, 15%–37%) [1]. Assuming a vaccination rate of 40.1% as the base-case value for our model, thus, leaves about 94% (1 − (0.401 × 0.15)) of hospital personnel susceptible to influenza infection. Our best-case assumption for sensitivity analysis was based on the effectiveness value of the influenza season 2011/2012 which was 49% (95% CI, 17%–49%) [26]. 

##### Secondary Attack Rate after Influenza Virus Transmission

Whilst the instantaneous risk of influenza transmission from an infective index case to a susceptible household contact has been estimated to be 0.32 person per day (95% CI, 0.26%–0.39%), irrespective of the manifestation of illness after infection [21], the secondary attack rate—defined as near contact with new onset of acute respiratory illness—was estimated at 20.2% (95% CI, 15.4%–25.6%), falling mostly within 3 to 7 days following symptom onset of the index case [33].

#### 2.3.2. Economic Parameters

##### Costs of the Rapid Influenza Test

According to the information provided by Quidel Inc. the costs of the Solana^®^ are uniformly €14 (without any volume discount). 

##### Costs of External PCR

A PCR for respiratory pathogens might be charged according to the positions 4780, 4782, 4783, and 4785 of the German Scale of Medical fees (GOÄ), which regulates the billing of medical services outside the statutory medical care in Germany. However, usually, significant discounts on official fee positions are agreed between external laboratory and the respective hospital. Taking into account the competition between laboratories, the actual prices vary between €30 and €65 (personal inquiry). As the mean value for our model, we considered the price of €44.88.

##### Hospital Opportunity Costs

Under the premise that most patients are accommodated in a twin-bedded room and that hospital wards in Germany are working at full capacity, the loss of the use of one bed is incurred by the hospital during the isolation period. New patients who could have been placed in the blocked beds are turned away. Consequently, the hospital will forego revenue (incur opportunity costs). These costs are not covered by healthcare insurance. To arrive at a representative estimate of the cost to hospitals of an unused bed, the minimal average revenue per bed and day was determined on the basis of the G-DRG catalog (version 2018, InEK GmbH, Siegburg, Germany). Using the DRG, the expenses for influenza patients without complicating pneumonia at the time of admission (G-DRG E79 C)—those who would have occupied a second now blocked bed in the twin-bedded room—are as follows:

Average daily reimbursement per bed = (Cost weight for G-DRG D62Z based on ICD-10 Code J10.1) × German base rate in 2019)/mean length of stay for G-DRG D62Z. Using the respective values as described in [18] and [34], this results in (€3544.97 × 0.461)/3.5 = €466.92.

Since the hospital also saves costs (variable costs) through the non-treatment of a new case, the amount of revenue loss was reduced by 25%, as suggested in hospital management theory [35], resulting in a value of €350.19 of opportunity costs per day. Opportunity costs might be stopped if a negative PCR result of the swab of an influenza suspect has been reported by the external laboratory to the hospital on the next day, or the two subsequent days.

##### Costs of Initial Treatment with Neuraminidase Inhibitors

As disease duration has been proven to be shortened by NI in some very large observational studies, it is recommended by the WHO [10], the European Centers for Disease Control (ECDC)/Centers for Disease Control and Prevention (CDC) [11], and Public Health England [12], that patients suspected or confirmed of having influenza and those who would be hospitalized, should be treated with NI. If there is no known resistance of the circulating viruses, Oseltavimir per os is recommend as the first-choice drug. It should be started as soon as possible in case of clinical suspicion, even without laboratory confirmation, because the biggest benefit can be expected within 48 hours, following onset of ILI symptoms. In addition, in hospitalized patients it is recommended by all three of the above-named institutions that treatment be initiated even at a later point, as evidence suggests benefit when initiation of treatment takes place up to 5 days following symptom onset. If the laboratory PCR result is negative, the antiviral therapy should be discontinued. The recommended dose of Oseltamivir is 75 mg orally, twice daily, over five days. A total of 75 mg can be administered by taking one 30 mg capsule plus a 45 mg capsule. The price of Oseltamivir per day can be calculated as follows—the price of 10 capsules of 30 mg is €24.26, that of 10 capsules of 45 mg is €37.59, Oseltamivir is considered to be administered twice daily for a period of 5 days, accordingly, the price per day is €61.85 divided by 5 (days) = €12.37 (Rote Liste^®^ 2019).

##### Revenue from Administering a Neuraminidase Inhibitor

Early administration of NI might reduce by one day the hospital stay of an influenza patient, although the diagnosis-related groups (DRG) flat reimbursement rate per influenza case stays the same. Additional revenue in favor of the hospital would be achieved by dividing the total DRG-revenue (€1,634.23) for a stay of 7 days for J10.1 patients, which the hospitals earns for the patient (see also 2.3) by 7 (i.e., €233.46), given that the patient would be treated in hospital for at least 2 days (minimum length of stay for a DRG service). Thus, under the assumption that a patient can be discharged one day earlier due to the clinical effect of the Oseltamivir treatment, the hospital saves €233.46. 

##### Costs of Intrahospital Transmission 

In cases of unidentified influenza without isolation, the hazard of influenza transmission to health care workers (HCW) through contact with an initially unidentified influenza patient, must be considered. In Germany, employees receive full salary during sick leave. An employed person who is sick with an uncomplicated influenza, will take an average of seven days off work (7.2 days in Germany [23] and in Norway [24].

Personnel costs were based on the average rates obtained from the most recent data (2017) of the German Federal Statistical Office (DESTATIS). According to [22], the gross annual earning of a hospital employee is €57,402. Divided by 365 days the loss for the hospital as an employer is €156.99 per day.

## 3. Results

In the base-case analysis, utilizing the Solana^®^ test in ILI patients is on average €132.61 less costly per eventually hospitalized patient, compared to the conventional clinical approach (see Table 2). Included in this amount is a cost saving of €6.90 in absolute terms per tested patient, in favor of the hospital. Above all, this is attributable to the principal advantage of the Solana^®^—that of recognizing influenza with high sensitivity—and to the corresponding early Oseltamivir treatment. 

Univariate sensitivity analysis, in which all variables in the decision trees receive the assigned values within their respective ranges, reveals that even a small decrease in influence in ILI patients (by 2.8% to 39.8%) results in a reversion of the absolute cost savings by utilizing the Solana^®^ as an add-on diagnostic test (Table 3). Potential cost savings also strongly depend on the amount of revenue the hospital gains per day if an influenza patient can be discharged earlier, than the fully paid revenue by the G-DRG flat rate. If the per-day revenue falls below €217.10, absolute cost savings can no longer be achieved with Solana^®^ testing. The same is true if the specificity of Solana^®^ falls below a threshold of 96.22%. 

The variation of the opportunity costs per day (blocked second bed in a twin-bed room) only has a marginal impact, as the threshold for reversion would only be achieved at actual opportunity costs of more than €402.37. Cost saving in absolute terms remain even when the values of the other model parameters are changed between plausible extremes. 

In probabilistic sensitivity analysis (PSA), i.e., under all reasonable assumptions, the costs of implementing the Solana^®^ for influenza testing at the expense of the hospital, on average amount to only €12.92 (Table 4). Here, absolute cost saving is no longer achieved but implementing the test does not exceed its purchasing costs. Of note, testing with Solana^®^ is constantly less expensive than the purely clinical approach and on average even less expensive than that in base analysis, with €144.13 (±€24.68) per tested patient, prior to hospitalization.

Following the test result of the Solana^®^, only 2.1% of the patients tested as a false positive and suffered from unnecessary isolation and Oseltamivir treatment for a total of 7 days, because no external PCR was performed. In contrast, according to the suggestions of conventional clinical judgement, the proportion of unnecessary bed blocking was more than thirteen-fold higher at 27.4%, although this mistake can be corrected when the result of the PCR is available after 1–2 days. Thus, the cost difference between the two strategies, with respect to opportunity costs—after exclusion of the costs of Solana^®^ and of the external PCR—is €75.42 (€128.2–€52.78) in favor of Solana^®^.

The costs of performing one external PCR in PSA are on average €46.66 per tested ILI patient, versus the costs of rapid testing of the Solana^®^ for which a retesting with PCR is not required, are €13.99. Thus, the lower costs for testing is immediately observable, with a difference of €32.67 per patient.

Due to the higher sensitivity of Solana^®^, more influenza patients could be properly treated with Oseltamivir than with the conventional strategy (32.2% versus 22.3%), resulting in a lower expenditure of €15.61.

Finally, lower sick day costs of €20.42 per tested patient, at the expenditure of the hospital, is incurred by using Solana^®^.

## 4. Discussion

Newer real-time tests such as the Solana^®^, showing both a sensitivity and a specificity exceeding 95%, come close to laboratory PCR test in their abilities to very rapidly confirm or exclude influenza. Therefore, they have the potential to either avoid unnecessary isolation—if influenza can largely be excluded—or to confirm that an isolation at the hospital ward is immediately required.

To date, the discussion on potential economic benefits of new rapid tests has focused on some aspects of resource management during the short duration of stay in the emergency rooms themselves. In a recently published model, Brachman et al. [36] describe that by testing 812 influenza suspects with the Alere^®^ i Influenza A&B test, during the influenza season 2016/2017, a total of 2733 hours could be saved and that this would be more than enough hours to give up space of a whole extra room in the emergency department. This room could then be used for other duties. However, the time portions that the authors added in their calculation (especially disinfection of multiple rooms including the X-ray suite and the examination room) are incidental and accrue at different points in time. Due to the fact that the working shifts of the personnel in the ER must always be fully paid—irrespective of the actual workload—the question arises as to how those minutes of time portions can be practically operationalized in order to gain reliable and reproducible effects. Other studies reported diminishing antibiotic prescriptions by using rapid tests prior to discharge, in hospitalized adults (e.g., Falsey et al. [37]), or in children presenting a pediatric emergency (e.g., Bonner et al. [38]). However, antibiotics prescribed in German emergency rooms for patients who would be discharged and sent home is paid by the PHI, not by the hospital, and are thus, not in the scope of our model. On the other hand, administering antibiotics in hospitalized patients can be guided by C-reactive protein (CRP)- or procalcitonin testing, which helps to differentiate between viral and bacterial infections. Accordingly, in two newer meta-analyses, the results on the reduction of the number of antibiotic prescriptions are heterogeneous [39,40].

The key to reduce costs by implementing a real-time influenza test from the hospital’s perspective, lies in the time lag between taking the swabs in the ER, which would in most cases be sent to an external laboratory, and receiving the PCR report one or two days later. PSA reveals that performing the Solana^®^ for influenza suspects leaving emergency rooms for admission to a German hospital ward is consistently less expensive than the conventional symptom-based judgement for which the PCR testing results is only available after 1 or 2 days of delay.

The majority of savings—€75.42 or 52.3% of the total amount of €144.13—are achieved through a reduction in unnecessary isolation days that comes about, as significantly fewer false assumptions are made regarding the presence of influenza in ILI patients. Each time a patient is wrongly assumed to be suffering from an influenza infection, a hospital bed is blocked and a hospital’s capacity reduced, leading to corresponding revenue loss for the hospital.

Those savings can be achieved in parallel with significantly lower testing costs where the difference of €32.67 per patient makes a contribution of 22.7% of the total cost saving.

Even more reduction in expenditures can be achieved if Oseltamivir can be promptly started in those patients who in fact have influenza. Patients placed on therapy immediately can usually be discharged 1 day earlier. Due to the higher sensitivity of the Solana^®^, the cost difference here was €15.61 per tested patient, which is equivalent to 10.8% of the total cost difference between Solana^®^, and the conventional approach. This improves the hospital´s reimbursement performance. 

A savings of €20.42 or 14.2% is made by the hospital in unrecognized and misclassified transmission by influenza patients (even on the very first day) to unvaccinated or ineffectively vaccinated and, hence, unprotected HCW. This amounts to a reduction in secondary cases and sick days that come at the expense of the hospital as an employer. This amount represents the lowest amount in cost-saving, relative to the conventional approach, due to our conservative assumption about five contacts susceptible to virus transmission is needed to produce one secondary influenza case. In reality, however, during the seasonal influenza, relatively more HCW in hospitals are verifiably infected by misclassified influenza patients. For example, Gianino et al. [41], in their most recently published survey on influenza-induced absenteeism, estimated costs of more than 1.7 million Euros per year, arising from one single large Italian hospital, but they assumed a vaccination coverage below 3%. In our model, we namely considered a much higher vaccination coverage of 41% of German HCW, but nevertheless, we might have underestimated the true number of secondary cases and the corresponding sick day costs. 

Paradoxically, and counter-intuitively, one strikingly favorable feature of the Solana^®^ has its drawbacks. The immediate detection of influenza due to its high sensitivity, because of which performing an external laboratory PCR is not required, brings additional costs that occur in those very low percentage of false-positive cases. Given an influenza prevalence of 0.33—the mean estimate of our PSA—and a proportion of 3.1% false positives with Solana^®^, about 1% of influenza suspects would unnecessarily be isolated for 7 days and would receive ineffective Oseltamivir treatment for the full course of five days, whilst in the conventional strategy the unnecessary isolation would be finished usually after one day, when the PCR report is available. 

Our study has some limitations that must be considered when interpreting our results. First, the general limitation of a single-center model deserves consideration. The use of extensive sensitivity analyses is an effort that addresses those limitations. However, to validate our estimates, more cost studies, preferably with a multicenter and prospective study design, are required.

Furthermore, our calculations refer only to hospitals that must send samples to an external laboratory for influenza testing and wait 1–2 days for the report, or to those hospitals in which test results from their own laboratories are delayed due to organizational reasons, i.e., due to prolonged sample transportation or lack of availability of laboratory staff during weekend or at night. These hospitals that have a laboratory department that already conducts high quality influenza tests at their disposal and quickly provide test reports, would not benefit in the manner described here.

Of note, any virus infection that can cause ILI symptoms at a severity level that leads to hospitalization should be considered a transmission risk. In particular, symptoms of RSV (respiratory syncytial virus) resemble those of influenza. RSV, like influenza, can spread and lead to pneumonia or exacerbation of chronic obstructive pulmonary disease, especially in older and immune-compromised patients. Including RSV as a parameter of the proposed real-time test regime in ER, could further enhance the accuracy of diagnosis and further reduce the number of unnecessary isolation cases and consequent in-hospital costs [42].

## 5. Conclusions

The utilization of the Solana^®^ test as an example for a new generation of rapid influenza tests is likely to reduce overall costs in cases of suspected influenza in German hospital emergency departments. As such, routine use of such rapid tests might also have a direct and positive impact on the control of influenza. Prospective clinical studies should be undertaken to further evaluate its economic advantages in the immediate future.

## Figures and Tables

**Table 1:** Input for cost–benefit analysis.

Variables Category	Variable Name	Distribution *	Value (Base Case)	Relative Change (Range)	Reference
Prevalence of influenza	Flu_prev	triangular	0.426	0.2–0.426	Calculated from [2]
Additional revenue per day due to NI	cRev_day	uniform	€233.46	±20% (€186.77–280.15	Calculated from InEK data [18]
Combined Solana^®^ specificity	Solana_spec	uniform	0.96.7	(95% CI) 0.954–0.977	[19]
Opportunity costs due to blocking twin bed	cOpp	triangular	€350.19	±20% (€280.15–€420.23)	Calculated from InEK data [18]
Probability of correctly excluding non-influenza	Clin_spec	uniform	0.601	95% CI (65.4–76.7)	[20]
Sensitivity of diagnosing influenza if present	Clin_sens	uniform	0.713	95% CI (0.65–76.7)	[20]
Costs of virustatics per day	cAntivir_day	triangular	€12.37	±20% (€9.89–€14.83)	Adapted from Rote Liste 2019
Costs of Solana^®^	cSolana	triangular	€14	±20% (€11.20–€16.80)	As declared by manufacturer
Combined sensitivity of the Solana^®^ test	Solana_sens	triangular	0.972	95% CI (0.954–0.984)	[19]
Secondary cases due to one unknown influenza case	sec_flu	normal	0.202	95% CI (0154–0.256)	[21]
Costs of productivity loss per day	cPL_day	triangular	€156.	±20% (€125.59–€188.39)	calculated from Federal Statistical Office data [22]
Number of days of HCW out of work due to influenza	sick_days	normal	7.2 10.32 days	±SD 8.9 days (5.76–8.64)	[23,24]
Probability of vaccinated HCW	pVacc_HCW	normal	0.401	±20% (0.33–0.49)	[25]
Probability that hospitalization is required	pHosp	uniform	0.18	0.157–0.23	Adapted from [1]
Costs of PCR in external laboratory	cPCR	uniform	€44.88	€30-65–€7.56	Nationwide laboratory inquiry
Effectiveness of influenza vaccination	Vacc_eff	uniform	0.15	0.15–0.49	[1,26]

* in probabilistic sensitivity analysis.

**Table 2:** Results of the base-case analysis.

Base-Case Analysis	Comparators	Mean Cost Per Patient (€)	Incremental Cost (€) *	Absolute Cost Savings (€)
ILI patients prior to hospitalisation followed by immediate intake of NI	Solana^®^ as an add-on	−6.90	0	−6.90
	Conventional approach	125.71	132.61	

* Incremental cost denotes the increase in total costs resulting from using the conventional approach alone versus including rapid testing.

**Table 3:** Tornado diagram * (real-time influenza testing versus the conventional clinical approach).

Variable Name	Variable Description	Variable Lowest Bound	Variable Highest Bound	Lowest cost Value	Highest Costs Value	Spread ^Ƭ^	Threshold Value ^µ^	Risk % ^¥^	Cumulative Risk%
Flu_prev	Prevalence of influenza	0.20	0.43	−6.90	48.19	55.09	<0.398	0.49	0.49
cRev_day	Additional revenue per day due to NI	186.77	280.15	−26.23	12.43	38.67	<217.10	0.24	0.74
Solana_spec	Combined Solana^®^ specificity	0.95	0.98	−21.32	11.86	33.18	<0.9622	0.18	0.92
cOpp	Opportunity costs due to blocking twin bed	280.15	420.23	−16.19	2.39	18.57	>402.37	0.06	0.97
cAntivir_day	Costs of virustatics per day	9.89	14.83	−12.27	−1.57	10.70	-	0.02	0.99
cSolana	Costs of Solana^®^	11.20	16.80	−9.70	−4.10	5.60	-	0.01	0.99
Solana_sens	Combined sensitivity of the Solana^®^ test	0.95	0.98	−8.87	−3.28	5.59	-	0.01	1.00
sec_flu	Secondary cases due to one unknown influenza case	0.15	0.26	−7.50	−6.21	1.29	-	0.00	1.00
cPL_day	Costs of productivity loss per day	125.59	188.39	−7.41	−6.39	1.02	-	0.00	1.00
sick_days	Number of days of HCW out of work due to influenza	5.76	8.64	−7.41	−6.39	1.02	-	0.00	1.00
Vacc_eff	Effectiveness of influenza vaccination	0.15	0.49	−7.28	−6.90	0.38	-	0.00	1.0
pVacc_HCW	Probability of HCW being vaccinated	0.33	0.49	−6.93	−6.86	0.07	-	0.00	1.00
pHosp	Probability that hospitalization is required	0.16	0.23	−6.90	−6.90	0.00	-	0.00	1.00
Clin_sens	Sensitivity of diagnosing influenza if present	0.65	0.77	−6.90	−6.90	0.00	-	0.00	1.00
Clin_spec	Probability of correctly excluding non-influenza	0.57	0.63	−6.90	−6.90	0.00	-	0.00	1.00
cPCR_ext	Costs of PCR in external laboratory	30.00	65.00	−6.90	−6.90	0.00		0.00	1.00

* One-way sensitivity analyses of all model variables arranged in order, with the variable with the biggest impact at the top and the variable with the smallest impact at the bottom; ^¥^ Risk%: This is a measure of how much of the total uncertainty is represented by the respective variable. The Risk% values sum to 1.0 across all the variables; ^Ƭ^ Highest cost value minus lowest cost value; ^µ^ Indicates the point at which absolute savings turn to expenditures. HCW: health care workers.

**Table 4:** Results of the probabilistic sensitivity analysis (Monte Carlo Simulation).

Probabilistic Sensitivity Analysis	Comparators	Mean Cost Per Patient (€)	Standard Deviation (± SD)	Incremental Cost (€) *
ILI patients prior to hospitalisation followed by immediate intake of NI	Solana^®^ as an add-on	12.92	24.66	0
	Conventional approach	157.05	24.68	144.13

* Incremental cost denotes the increase in total costs resulting from using the conventional approach alone versus including rapid testing.
